# Analysis and Modeling of Realistic Compound Channels in Transparent Relay Transmissions

**DOI:** 10.1155/2014/538578

**Published:** 2014-02-18

**Authors:** Cibile K. Kanjirathumkal, Sameer S. Mohammed, Lillykutty Jacob

**Affiliations:** National Institute of Technology Calicut, Calicut 673601, India

## Abstract

Analytical approaches for the characterisation of the compound channels in transparent multihop relay transmissions over independent fading channels are considered in this paper. Compound channels with homogeneous links are considered first. Using Mellin transform technique, exact expressions are derived for the moments of cascaded Weibull distributions. Subsequently, two performance metrics, namely, *coefficient of variation* and *amount of fade*, are derived using the computed moments. These metrics quantify the possible variations in the channel gain and signal to noise ratio from their respective average values and can be used to characterise the achievable receiver performance. This approach is suitable for analysing more realistic compound channel models for scattering density variations of the environment, experienced in multihop relay transmissions. The performance metrics for such *heterogeneous compound channels* having distinct distribution in each hop are computed and compared with those having identical constituent component distributions. The moments and the coefficient of variation computed are then used to develop computationally efficient estimators for the distribution parameters and the optimal hop count. The metrics and estimators proposed are complemented with numerical and simulation results to demonstrate the impact of the accuracy of the approaches.

## 1. Introduction

Precise channel models are of utmost importance in the design of wireless systems, as they influence power budgeting, transceiver design, performance prediction, and so forth, of the overall system. Cooperative relaying techniques enable end-to-end network connectivity where traditional direct link architectures are impractical due to location constraints. In multihop cooperative relay communication, precise statistical models for the compound channels between the source-destination pair are unknown in some cases and too complex for tractable performance analysis in other cases. In cooperative multihop diversity networks, the possibility of occurrence of distinct component distributions for the individual hops of multihop transmission cannot be neglected, for example, the situation where the source-relay link follows one distribution (say Rayleigh) while relay-destination link follows another distribution (Rician) due to the power boosting being done at relay node. The primary objective of this paper is to characterize cascaded fading channels so that multihop cooperative transparent or non-regenerative relay communication can be designed to achieve their preset goals.

Uncertainty analyses of multihop cooperative communication under consideration can be accomplished by its distribution, statistical moments such as mean, variance, and other higher order moments. In most cases, the derivation of exact probability density function (pdf) is a difficult task, if not impossible because of the product forms involved in distributions. In many cases, it is sufficient to estimate the statistical moments to have an assessment of the system. There are only a few channel models and metrics available for characterising cascaded multihop relay transmissions as compared to direct link wireless systems. In diversity combining receivers, optimum performance is achieved by selecting the branches with minimum symbol error rate (SER). Such technique requires continuous estimation of the SER, leading to evaluation of high complexity expressions or closed form approximations for the SER calculations. Under deep fades, the conventional performance measures like average SER and outage probability used in direct link cellular system analysis may not necessarily reflect the fading effects in the constituent channels. To combat the fading effect, associated use of alternate paths are exploited in a diversity system like cooperative communication network. Knowledge of higher order moments and reliable performance metrics is vital for improving the dynamic range of receivers in such communication systems. Coefficient of variation (CV) [1] and amount of fade (AF) are the two ratio measures used for assessments under highly unpredictable statistical scenarios where increase in transmitter power cannot reduce fading induced fluctuations. These measures incorporate the variation in channel gain and variation in signal to noise ratio (SNR), of the end to end channel that is compound in nature due to multiplicative effects. They can be used to fix the detector voltage level threshold and dynamic range of the receiver measurement unit.

Parametrisation of the approximated empirical distribution at the receiver can also be done with the CV measure, for a known family of cascaded distributions by using estimators based on method of moments (MOM). Further, CV can be used for estimating the hop count, by which the fundamental question of how to select the best order of cascade for a particular terrain from a given set of measurement data can be dealt with.

Generation of moments from first kind characteristic function for the compound channel models often require, solutions of incomplete integrals. Alternately, characteristic function of the second kind, derived using Mellin transform, can be used to find the stochastic properties of nonnegative random variables [1]. The Mellin transform approach [2] is used for deriving the moments of the cascaded channels so that it can be conveniently extended to various combinations of component channels in a compound channel model. Though Mellin transform approach had been used in many of the previous works, the explicit use of its properties to find the moments of SNR, especially for *heterogeneous compound channels*, is not exploited yet. The convenience in using Mellin transform is that it converts the exponentials to polynomials so that convolution theorem can be applied easily for the multiplicative scenarios. Another aspect is that the computations of moments or the metrics do not require the probability density function (pdf) of the end-to-end compound channel, where as the derived moments can be used to find the pdf of the end to end channel. Weibull distribution [3] can be accounted as a terrain specific distribution providing greater flexibility in describing the fading severity of a channel. This is selected as a representative distribution for illustrating the approach, as the exponentiated random variable (RV) is powered by the fading parameter of the distribution. The analyses are then conveniently extended to Rayleigh distribution and their combinations also. For Nakagami-*m* distribution, the exponentiated RV is getting powered by two. Hence, extension of the approach to other combinations of terrain specific distributions like Nakagami-*m*, Gamma, and so forth (for which Mellin transform exists) can be done easily.

In order to find the exact moments, the major challenge is to find a proper variable transformation rule to represent the overall pdf in a tractable and compact form, especially in heterogeneous compound channel cases. Alternately, the proposed method provides a feasible solution to this issue, by using the product convolution property of Mellin transform, without the knowledge of overall pdf.

Our major contributions in brief are listed here. (i) An approach to analyse a heterogeneous compound channel model that can precisely depict the heterogeneous scattering environment for a multihop wireless communication system is proposed. (ii) Exact expressions for moments of overall distribution and performance metrics such as CV and AF for such compound channels are derived. (iii) A comparison study of these measures with that of homogeneous distributions is performed. (vi) Estimators for finding distribution parameters (shape and scale parameters) of *N*-Weibull distributions are presented.

The rest of the paper is organised as follows.  Section 2 presents the closely related works. Two cases of compound channel models for a multihop relay network are presented in Section 3.  Section 4 deals with the computation of moments. Using the computed moments, exact expressions for performance metrics are derived in Section 5, where the discussions on the results are also included. Moment based estimators for distribution parameters are presented with results in Section 6, and Section 7 concludes the paper.

## 2. Related Works

Most of the previous works on the product of independent random variables deal with product pdf computation based on the moments generated by random variable exponentiation. In [4, 5] the pdf for the product of independent random variables was derived in terms of the H-function, which includes most of the commonly used distributions as special cases. In [6], the pdf for the product of Rayleigh random variables was derived in terms of the Meijer-G function, as well as in terms of infinite series. An approximate product pdf expression for various identical distributions is derived in [7], and good accuracy is achieved in comparison to exact expression by incorporating approximation to the transform integral. Using approximations for the exponential moment generating function, BER expressions are derived in [8] for the product of K-function distributions. The product pdf for Weibull distribution in [9] uses central limit theorem to the logarithm of a product of a large number of RVs, where the basic Weibull pdf is used instead of the generic Weibull pdf that is considered in our proposed work. In [10], the end to end SNR of Weibull distributed cascaded channel is computed using Pade approximant method. In this case also, the density function used is specific for the given relationship between the scale and shape parameters. Performance metrics for homogeneous cascaded Weibull distribution are considered in [1]. Despite these contributions, a unified characteristic function based method for the computation of exact moments and performance measures, which can be conveniently extended to other combinations of distinct component distributions, is not available in the literature. An estimator for channel parameters, with simple implementation, based on method of moments is proposed and the fundamental question of how to select the best order of cascade *N* for a particular topology, for a given set of measurement data, is also dealt with.

## 3. Compound Channel Model

We focus on quantifying the end to end performance of multihop relay transmissions in the context of collaborative/cooperative wireless communication systems, where relaying is used to overcome the effects of highly shadowed or deeply faded links. In particular, we consider one multihop branch of the multihop, multibranch cooperative diversity network. A multihop branch with *N* − 1 serial relays in between the transmitter and receiver (destination) is considered.

For such a system, with RV*W* representing the compound channel gain and *N* independent RV*sX*_*i*_ representing the individual channel gains, we have

(1) $$\begin{array}{c} W=\prod_{i=1}^{N}X_{i}, \end{array}$$

where *X*_*i*_'s are non negative RV*s* that can have any distribution depending on the nature of the radio propagation environment.

We consider two types of compound channels: assuming Weibull distribution on individual component channels but with variable scaling factors for the components, we can effectively model *W* as a single product distribution, which we refer to as homogeneous compound channel case. Weibull distribution that efficiently depict the amplitude and power statistics of a channel in highly unpredictable scenarios is selected for the component channels [11]:

(2) fXi(x;α,β)=βα(xα)β−1e−(x/α)βU(x); α>0,  β>0,

where *U*(*x*) is unit step function, *α* is the scale parameter related to nonlinearity of the environment, and *β* is the shape parameter which alters distribution properties more fundamentally than the scale parameter. In this modified expression, the exponentiated scaled RV is raised to a power equal to the shape parameter *β*. Rayleigh and exponential distributions can be obtained from this Weibull distribution by setting *β* = 2 and *β* = 1, respectively.

As mentioned earlier, the assumption of homogeneous scattering environment for all the constituent links is definitely an approximation. In many practical scenarios, cascaded channel is often characterised by heterogeneous environments due to scattering density variations.

**Figure 1:**
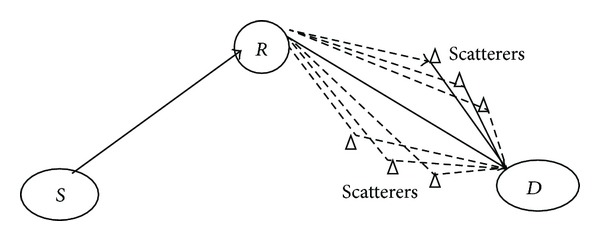
*Heterogeneous compound channel* formation due to environmental (scattering) variations in each hop.

Figure 1 explains this scenario with a two-hop system where the relay to destination link experiences heavy scattering density as compared to the source to relay link, forming a heterogeneous compound channel of Weibull × Rayleigh distributions. This is the second type of compound channel which is referred to as heterogeneous compound channel case. Using the Mellin transform of Nakagami-*m* distribution, fading statistics of other combinations like Weibull × Nakagami-*m*, Rayleigh × Weibull × Nakagami-*m*, and so forth can be obtained.

## 4. Computation of Moments for Compound Channels

We propose the use of second kind characteristic function [12], based on Mellin transform, to reduce the complexity associated with compound channel modelling. The main advantage of the proposed technique is that, instead of exponentiation of RV*s*, only powers of them are required for computation of moments. This approach can readily be applied to find the moments of compound channel with distinct component distributions in a simple and tractable form. Since the approach is to be extended to other terrain specific distributions also, a short description of homogeneous case considered in [1] is given below.

### 4.1. Homogeneous Case

In the case of Weibull distribution, the *p* ^th^ moment becomes

(3) $$\begin{array}{c} m_{p}=\int_{0}^{\infty }x^{p}\frac{\beta }{\alpha }{\left(\frac{x}{\alpha }\right)}^{\beta -1}e^{-{\left(x/\alpha \right)}^{\beta }}dx. \end{array}$$

With proper variable substitution and reduction techniques [1], this can be expressed in a compact form in terms of gamma function as

(4) $$\begin{array}{c} m_{p}=\alpha^{p}\Gamma \left(1+\frac{p}{\beta }\right), \end{array}$$

where Γ(*t*) = ∫_0_^*∞*^ *x* ^*t*−1^ *e* ^−*x*^ *dx*. Considering the multiplicative effects in a multihop branch, the moments of the compound channel gain *W* are given by

(5) $$\begin{array}{c} m_{p_{N}}=E\left[{\left(\prod_{i=1}^{N}X_{i}\right)}^{p}\right]=\alpha^{Np}\left[\Gamma^{N}\left(1+\frac{p}{\beta }\right)\right], \end{array}$$

where *X*_*i*_'s have independent and identical distributions because of the homogeneous assumption. However, proper variable transformations are often quite tedious and moment computations by the above conventional method become complex. Alternately, using Mellin transform and its properties, such calculations can be made simpler, resulting in compact expressions even for nonidentical, dissimilar component distributions. The Mellin transform of a probability density function *f*_*X*_*i*__(*x*) is given as [13]

(6) $$\begin{array}{c} M\left(f_{X_{i}}\left(x\right),s\right)=\int_{0}^{\infty }x^{s-1}f_{X_{i}}\left(x\right)dx, \end{array}$$

where *s* = *a* + *jb* ∈ *C* is a complex transform variable. By setting *s* = *p* + 1, moments can be generated from (6) as

(7) $$\begin{array}{c} m_{p}=M{\left[\left(f_{X_{i}}\left(x\right),s\right)\right]}_{s=p+1}. \end{array}$$

Using (2) and (6), the Mellin transform of Weibull distribution can be written as

(8) $$\begin{array}{c} M\left(f_{X_{i}}\left(x\right),s\right)=\frac{\beta }{\alpha^{\beta }}\int_{0}^{\infty }x^{(s+\beta -1)-1}e^{-{\left(x/\alpha \right)}^{\beta }}dx. \end{array}$$

Using certain properties of the Mellin transform pairs [1, 13], we get the transform in terms of the gamma function as

(9) $$\begin{array}{c} M\left(f_{X_{i}}\left(x\right),s\right)=\frac{1}{\alpha^{\beta }}\alpha^{s+t}\Gamma \left(\frac{s+t}{\beta }\right), \end{array}$$

where *t* = *β* − 1. This transform operator represents the second kind characteristic function *Q*__2_*X*_(*s*) of the Weibull distribution expressed in a general compact form as

(10) $$\begin{array}{c} Q_{2X}\left(s\right)=M\left[f_{X_{i}}\left(x\right),s\right]=\alpha^{s-1}\Gamma \left(\frac{s+\beta -1}{\beta }\right). \end{array}$$

Therefore, moments of Weibull distribution can be obtained from (10) by setting *s* = *p* + 1; that is,

(11) $$\begin{array}{c} m_{p}=Q_{{\;}_{2}X}\left(s\right){|}_{s=p+1}=\alpha^{p}\Gamma \left(\frac{p+\beta }{\beta }\right). \end{array}$$

For the computation of the moments of a dual hop compound channel pdf, the usual techniques of conditioning on RV*s* or Jacobian transformations are no longer required if Mellin transform properties are exploited. The Mellin convolution between the pdfs *f*_X_1__(*x*_1_) and *f*_*X*_2__(*x*_2_) can be extended to *N* number of pdfs. Therefore, Mellin transform of the compound pdf of an *N*-hop cascaded branch, in terms of the transforms of the component channel pdfs, is given by

(12) $$\begin{array}{c} M\left[\left(f_{W}\left(w\right),s\right)\right]=\prod_{i=1}^{N}M\left[\left(f_{{\text{X}}_{i}}\left(x_{i}\right),s\right)\right]=Q_{{\;}_{2}W}\left(s\right). \end{array}$$

Assuming identical parameters for the component distributions, the transform of the compound pdf is obtained as

(13) $$\begin{array}{c} M\left[\left(f_{W}\left(w\right),s\right)\right]=\alpha^{(s-1)N}\left[\Gamma^{N}\left(\frac{s+\beta -1}{\beta }\right)\right]. \end{array}$$

This being the second kind characteristic function of the compound pdf, we can find the various first kind moments of the compound channel gain by setting *s* = *p* + 1:

(14) $$\begin{array}{c} m_{p_{N}}=E\left[W^{p}\right]=\alpha^{Np}\left[\Gamma^{N}\left(1+\frac{p}{\beta }\right)\right]. \end{array}$$

This is the same as (5) obtained using conventional method, where the proper variable transformations are often quite tedious. These results are very useful in practical scenarios, where the distribution parameters are estimated from the various sample moments obtained from the collected data.

Similarly, extending the above approach to Nakagami-*m* distribution, the *p* ^th^ moment of *N*-cascaded compound channel can be derived as

(15) $$\begin{array}{c} m_{pN}=E\left[Y^{p}\right]=\frac{\Gamma^{N}\left(m+p/2\right)}{\Gamma^{N}\left(m\right)}{\left(\frac{\Omega }{m}\right)}^{pN/2}, \end{array}$$

where *Y* represents the product RV of Nakagami-*m* distributions having scaling factor *Ω* and fading figure *m*.

### 4.2. Heterogeneous Case

The environmental effects like variation in local scattering density as described in Figure 1 can result in RV*s* having distinct density function for each hop. If Weibull distribution is used for approximate model, the order to which the exponentiated RV is raised for each component channel will be different. The average power or the scaling will also be different in each component channel. For an accurate model, each channel needs to be characterized with separate distribution and not as special cases of one distribution. Computation of compound channel pdf using the conventional method of successive application of Jacobian transformation or conditioning of RV*s* becomes difficult as the number of hops *N* becomes larger, even for the case of same distribution family but with nonidentical parameters. In the case of distinct or nonidentical distributions, this conventional method may lead to intractable mathematical formulations. But it is possible to derive the moments using the proposed Mellin transform approach in a convenient way. Consider a dual hop compound channel with one hop characterised by Weibull distribution and the other by Rayleigh distribution with pdf given by

(16) $$\begin{array}{c} f_{R}\left(x;\alpha_{1}\right)=2\left(\frac{x}{\alpha_{1}^{2}}\right)\;e^{-{\left(x/\alpha_{1}\right)}^{2}}U\left(x\right), \end{array}$$

where *α*_1_^2^/2 denotes the variance. The Mellin transform of Rayleigh distribution is given by

(17) $$\begin{array}{c} M\left[\left(f_{R}\left(x\right),s\right)\right]=\alpha_{1}^{s-1}\Gamma \left(\frac{s+1}{2}\right). \end{array}$$

Let *H* represents the heterogeneous compound channel gain. Using the individual Mellin transforms of distinct distributions and the product convolution property of Mellin transform, the transform of the heterogeneous compound pdf, *f*_*H*_(*h*), is

(18) $$\begin{array}{c} M\left[\left(f_{H}\left(h\right),s\right)\right]=\alpha_{1}^{s-1}\Gamma \left(\frac{s+1}{2}\right)\alpha_{2}^{s-1}\Gamma \left(\frac{s+\beta -1}{\beta }\right). \end{array}$$

Setting *s* = *p* + 1 in this second kind characteristic function of the compound channel, the exact moments for such a heterogeneous channel can be obtained as

(19) $$\begin{array}{c} m_{p}=\alpha_{1}^{p}\Gamma \left(\frac{p+2}{2}\right)\alpha_{2}^{p}\Gamma \left(1+\frac{p}{\beta }\right). \end{array}$$

This can be extended to other combinations of distributions and also to different number of hops. In conventional approaches, moments are found from the moment generating function (MGF), which requires the computation of overall pdf. However, in many cases, the compound channel pdf for nonidentical and heterogeneous cases will not be in a mathematically tractable form to compute the MGF of the overall channel, and hence approximations are to be made for. Whereas in our approach, since the component channel distributions are expressed in the transform domain itself, the involved mathematical complexity is reduced.

## 5. Performance Metrics for Compound Channels

Performance measures based on moments can be effectively used to characterize the channel in the context of cooperative relay communication systems. The data received over a compound channel with widely dispersed distribution is more difficult to detect. The possible deviation of the data from the average value can be used as a critical performance measure. We derive two such measures for the compound channels, which are useful for capturing the effect of fading induced fluctuations that cannot be compensated just by increasing the transmitted power. Here we derive two important parameters that are capable of providing deep insight into the channel pdfs.

### 5.1. Coefficient of Variation (CV)

Coefficient of variation is a normalized measure of dispersion of the probability distribution and is defined as the ratio of standard deviation (SD) to mean (*μ*). Considering the attenuation effect on data signal by channel gain, CV is a useful metric to analyse the range of variation of signal relative to the mean size of the observation. During the design and validation of the receiver unit, an assessment of data variability by measures such as SD or CV is critical in determining whether the signal can be detected within the specified confidence interval. In many comparison applications, SD becomes meaningless as it depends on the units of variables and the mean values about which they occur. Since CV is a unitless measure, the regularity/variability of the channel induced fading effects at signal level can be assessed effectively. By this method, the characterisation of individual branches of a multibranch multihop transmission system becomes more reliable and accurate.

#### 5.1.1. Homogeneous Case

In a multihop transparent relay network, the transmitted signal as well as the noise will be experiencing cumulative multiplication by the channel gain of the constituent links. The CV measure is quite useful in this scenario as the standard deviation must always be understood in the context of the mean of the data. Using the moments computed by (14) in Section 4.1 for the homogeneous compound channel with *N* identical Weibull distributions, the mean *μ* and variance *ν* of the compound channel gain *W* can be readily computed as

(20) $$\begin{array}{c} \mu =\alpha^{N}\left[\Gamma^{N}\left(1+\frac{1}{\beta }\right)\right],\\ \nu =\alpha^{2N}\left[\Gamma^{N}\left(1+\frac{2}{\beta }\right)\right]-\mu^{2}. \end{array}$$

Hence CV is

(21) $$\begin{array}{c} \text{CV}=\frac{\sqrt{\alpha^{2N}\Gamma^{N}\left(1+2/\beta \right)-\alpha^{2N}\Gamma^{2N}\left(1+1/\beta \right)}}{\alpha^{N}\Gamma^{N}\left(1+1/\beta \right)}. \end{array}$$

**Figure 2:**
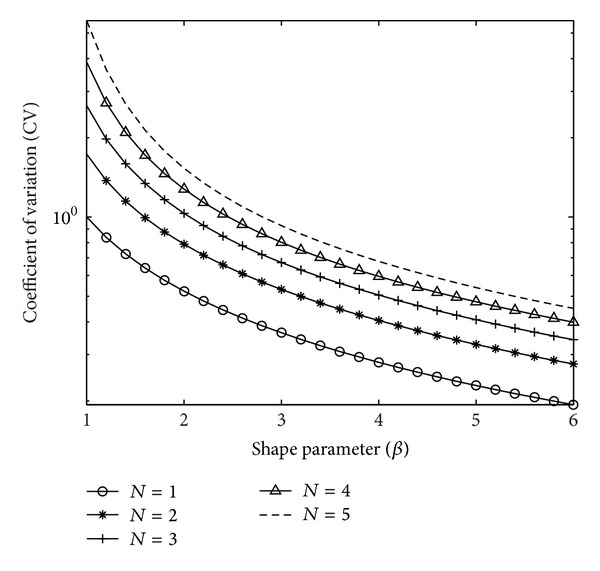
Coefficient of variation against *β* for various number of hops.

The coefficient of variation as a function of the number of hops *N* and the shape parameter for fading severity *β* is plotted in Figure 2. The channel for which the CV is large indicates that the channel gains have more variability. As *β* increases, the fading severity decreases, resulting in reduced variability for the channel coefficients and hence CV measure also decreases. For a single hop, CV shows the least measure for all values of *β*, indicating more regularity of the distribution function, where as the CV measure for *N* = 5 denotes less regular distributions due to cascading effects. It is to be noted that the CV becomes equal to unity, for *N* = 1 and *β* = 1, as the distribution reduces to exponential, and the CV for exponential distribution is unity. Similarly, for *N* = 1 and *β* = 2, CV = 0.5227, which is the CV measure of a Rayleigh distribution. This shows the accuracy of the general expressions we have derived for *N*-Weibull distributions. In this way, based on the CV value at the receiver, computed from sample moments, the distributions can be identified and hence outage probability can be derived. Thus, a comparison of variability of two or more branches (of possibly varying hop counts) of a multihop multibranch cooperative communication system can be done based on these graphs.

#### 5.1.2. Heterogeneous Case

As mentioned earlier, in the study of multiple scattering in mobile radio channels, the environmental conditions producing severe fading need to be considered and must be modelled properly. More importantly, situations are encountered for which none of the known distributions seem to adequately fit experimental data [11]. The exact pdf of a heterogeneous compound channel with distinct distributions (e.g., Weibull × Rayleigh) is not available in simple mathematical functions but involves special functions. As we have shown earlier, for our proposed Mellin transform based approach, only the transforms of the individual component channels are required instead of the compound channel pdf for computing the overall moments. Using the moments given by (19) and denoting *α*_1_ *α*_2_ = *α*, the expected value for the two-hop Rayleigh × Weibull heterogeneous channel is given by

(22) $$\begin{array}{c} m_{1}=\alpha \frac{\sqrt{\pi }}{2}\Gamma \left(1+\frac{1}{\beta }\right). \end{array}$$

Second moment of the channel is given by

(23) $$\begin{array}{c} m_{2}=\alpha^{2}\Gamma \left(1+\frac{2}{\beta }\right) \end{array}$$

from which the variance *ν* and coefficient of variance CV are computed as

(24) $$\begin{array}{c} \nu =\alpha^{2}\Gamma \left(1+\frac{2}{\beta }\right)-\frac{\pi }{4}\alpha^{2}\Gamma^{2}\left(1+\frac{1}{\beta }\right),\\ \text{CV}=\sqrt{\frac{4\Gamma \left(1+2/\beta \right)}{\pi \Gamma^{2}\left(1+1/\beta \right)}-1}. \end{array}$$

**Figure 3:**
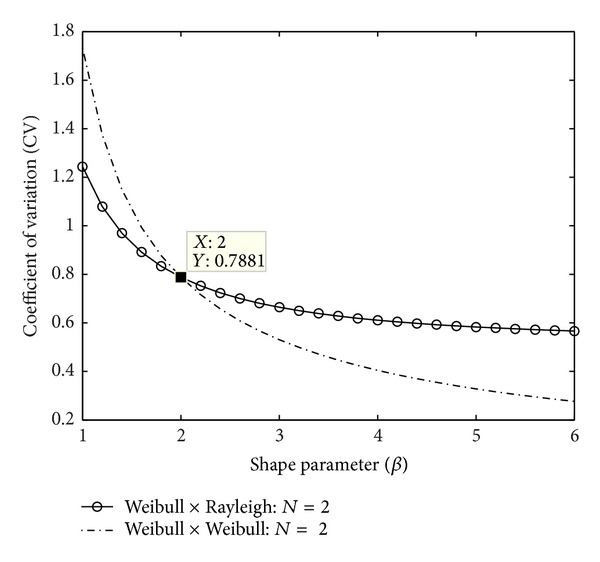
Channel variability comparison for *heterogeneous and homogeneous compound channels. *

Figure 3 displays a comparison of distribution variability of a homogeneous and a heterogeneous compound channel with *N* = 2 for various shape parameter (*β*) values. This inference regarding the variability of channel gain with respect to *β* may not be obviously obtained from a pdf plot of the distributions. It may be noted that when *β* = 2, the heterogeneous channel becomes homogeneous two-Rayleigh channel with CV = 0.7881, which is the same value that we get from the general expression for CV for *N*-Weibull distribution for *N* = 2. A lower value of CV for a distribution translates to statistical robustness and good SNR as the dispersion is small. Note that the homogeneous compound channel is more regular (lower CV value) for *β* > 2, and, for *β* ≤ 2, it is the heterogeneous compound channel that is more regular. This is due to the fact that, for larger *β* values, fading is less for Weibull distributions and the expected values become exponential in nature for a Weibull × Weibull distribution. This results in reduced CV values. But for smaller values of *β*, channel gain fading severity is more for a Weibull distribution, while Rayleigh distribution is independent of *β* variations. So the effect of channel attenuation is reflected as fixed scaling to the Weibull distribution in this region.

### 5.2. Amount of Fade (AF)

In a diversity combining system, the distribution of received SNR is of primary importance than the received signal distribution. A performance measure that is utilised to parametrise SNR distribution is more suitable than other types of performance measures [14]. AF is a unified measure of severity of SNR fading in a communication system, defined as [15, 16]

(25) $$\begin{array}{c} \text{AF}=\frac{\text{var}\left(Z^{2}\right)}{{\left(E\left[Z\right]\right)}^{2}}, \end{array}$$

where *Z* is the instantaneous SNR which can be expressed in terms of the channel gain *X* and SNR per bit, *E*_*b*_/*N*_0_, as

(26) $$\begin{array}{c} Z=X^{2}\frac{E_{b}}{N_{0}}, \end{array}$$

where *E*_*b*_ is the average energy of the transmitted symbol (bit) and *N*_0_ is the additive white Gaussian noise power spectral density. To compute the statistical moments of SNR, a pdf transformation according to (26) is required. Using the transformed pdf and the Mellin transform approach through (6)–(9), for the Weibull distribution that we have considered, the expression for AF can be derived as follows: (since AF is the ratio given by (25), *E*_*b*_/*N*_0_ is dropped in the derivation).

#### 5.2.1. Homogeneous Case

By applying the pdf transformation rule [14] to the given generic-Weibull pdf, the SNR distribution of each channel is derived as

(27) $$\begin{array}{c} f_{Z}\left(z\right)=\frac{\beta }{2\alpha^{2}}{\left(\frac{z}{\alpha^{2}}\right)}^{\beta /2-1}\;e^{-{\left(z/\alpha^{2}\right)}^{\beta /2}}. \end{array}$$

Mellin transform of this is obtained as

(28) $$\begin{array}{c} M\left(f_{Z}\left(z\right),s\right)=\alpha^{2(s-1)}\Gamma \left(\frac{2s+\beta -2}{\beta }\right). \end{array}$$

Using the variable transformation *s* = *p* + 1, the *p*th order moment for the *Z* is

(29) $$\begin{array}{c} m_{p}\left(z\right)=E\left[Z^{p}\right]=\alpha^{2p}\Gamma \left(1+\frac{2p}{\beta }\right). \end{array}$$

Defining *γ* as the end to end SNR, given by

(30) $$\begin{array}{c} \gamma =\prod_{i=1}^{N}X_{i}^{2}\frac{E_{b}}{N_{0}}, \end{array}$$

the moments for the end to end SNR, *E*[*γ* ^*p*^], are given by

(31) $$\begin{array}{c} m_{p}\left(\gamma \right)=\alpha^{2pN}\Gamma^{N}\left(1+\frac{2p}{\beta }\right). \end{array}$$

Substituting *p* = 1 and *p* = 2, respectively, in (31), the first and second order moments of SNR are computed as

(32) $$\begin{array}{c} E\left[\gamma \right]=\alpha^{2N}\Gamma^{N}\left(1+\frac{2}{\beta }\right),\\ E\left[\gamma^{2}\right]=\alpha^{4N}\Gamma^{N}\left(1+\frac{4}{\beta }\right). \end{array}$$

Using the definition for AF from (25) the *AF* for the *N*-Weibull compound channel can be obtained as

(33) $$\begin{array}{c} AF=\frac{\Gamma^{N}\left(1+4/\beta \right)}{\Gamma^{2N}\left(1+2/\beta \right)}-1. \end{array}$$

**Figure 4:**
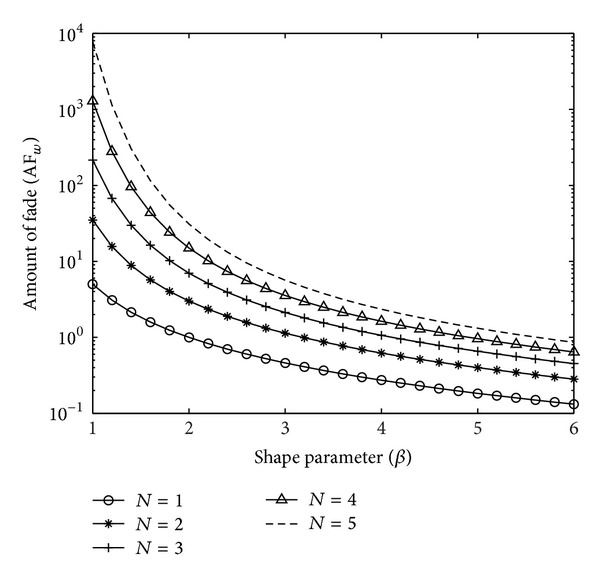
Amount of fade for *N*-Weibull compound channel.

The expression reveals the dependency of SNR fading severity on the shape parameter *β* and the number of hops *N*. A larger value indicates more severe fading, which translates to greater degradation in system performance [17]. The computation of AF for compound channels lends insights to the changes in the average SNR distribution due to multiplicative effects in *N*-hop systems. In a multibranch switched diversity system [18], this value can be used to track the diversity branch quality.  Figure 4 explains the variations in SNR (in terms of AF) for the *N*-Weibull homogeneous compound channel, with respect to *β*, for various hop counts. As the number of hops increases, the AF also increases indicating fading severity experienced for the Weibull compound channel due to cascading. Note that, being a ratio, *AF* is independent of the parameter *α* of the Weibull distribution, though the various moments depend on it.

#### 5.2.2. Heterogeneous Case

The novelty and convenience of our approach is explicit in the computation of the moments of SNR distribution of heterogeneous channel case. Instead of using the pdf transform approach, the general techniques available with Mellin transform approach for variable transformation can be conveniently applied to find the moments of the SNR distribution. The Mellin transform of the transformed variable *Z*, *M*(*f*_*Z*_(*z*), *s*), denoted as *M*_*Z*_(*s*), can be conveniently computed as

(34) $$\begin{array}{c} M_{Z}\left(s\right)=E\left[Z^{s-1}\right]=M_{X}\left(2s-1\right). \end{array}$$

Using this technique, for the given Weibull × Rayleigh heterogeneous channel, the moments of SNR distribution can be obtained as

(35) $$\begin{array}{c} m_{p}=\alpha^{2p}\Gamma \left(1+p\right)\Gamma \left(1+\frac{2p}{\beta }\right) \end{array}$$

from which first and second moments of the SNR distribution are, respectively,

(36) $$\begin{array}{c} E\left[\gamma \right]=\alpha^{2}\Gamma \left(2\right)\Gamma \left(1+\frac{2}{\beta }\right),\\ E\left[\gamma^{2}\right]=\alpha^{4}\Gamma \left(3\right)\Gamma \left(1+\frac{4}{\beta }\right). \end{array}$$

**Figure 5:**
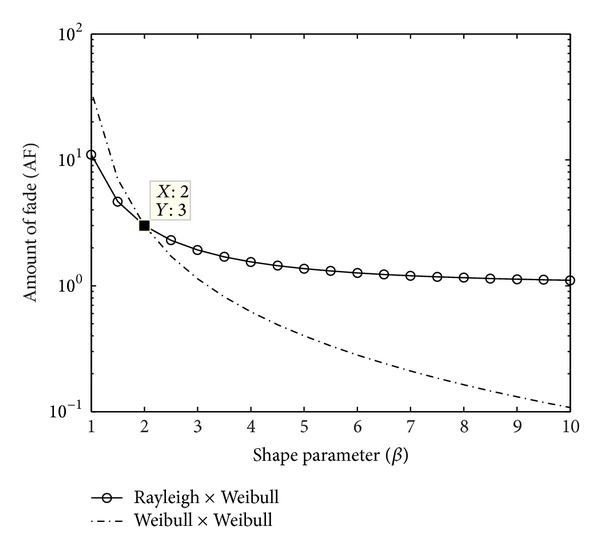
Amount of fade for Rayleigh × Weibull *heterogeneous compound channel. *

Therefore, using the gamma function identity Γ(3) = 2, the *AF* for Weibull × Rayleigh channel is given by

(37) $$\begin{array}{c} AF=2\frac{\Gamma \left(1+4/\beta \right)}{\Gamma^{2}\left(1+4/\beta \right)}-1 \end{array}$$

and is plotted in Figure 5.

Also, plotted in this figure is the two-hop Weibull homogeneous case for comparison. Compared to pdf,more inferences regarding the SNR statistics with respect to *β* variation can be obtained from this figure. It may be noted that the rate of decrease of AF with respect to *β* is smaller for the heterogeneous channel. This is due to the fact that the amplitude distribution of Rayleigh is independent of *β* variations. But, for two-Weibull distribution, the variability of average SNR is less (lower AF value) for higher values of *β* as the attenuation gets reduced in this region. At *β* = 2, the two component channels become the same and the compound channel becomes product of two Rayleigh channels. We get *AF* = 3 for *β* = 2 from the graph, which is equal to the value reported in [6] where the AF for *N*-Rayleigh compound channel is given as equal to 2^*N*^ − 1.

## 6. Coefficient of Variation: Applications 

Two applications of CV in the context of multihop compound channel are discussed in this section. The first one is the use of CV and moments in the parametrisation of the empirical distribution function defined by the collected data. Second one is the use of CV in estimating the number of hops for a multihop that can be supported by the destination receiver for a given standard deviation of the collected data.

### 6.1. Estimators for the Channel Parameters

Given a set of observations, an important task is the calibration of the distribution parameters so that the samples can be properly represented. In many such cases, maximum likelihood estimator (MLE) is typically considered as the best possible technique for estimating the channel parameters. However, MLE requires the knowledge of pdf in simple mathematical functions. But for a cascaded multihop scenario, exact pdf is normally available in the form of special functions like Meijer-G or H-functions only. Method of moments [19] can provide an estimator for distribution parameters, which is easy to determine and simple to implement, without using the pdf. 

In the method of moments approach, the *p*th order moments (for different values of *p*) of the distribution whose parameters are to be estimated are equated to the corresponding empirical moments of the observed data. As higher order moments are exceedingly difficult to be estimated accurately due to their large variance, a computationally efficient technique based on method of moments, using only lower order moments, is developed. This can be used to estimate the parameters of a broad class of compound channel models of varying cascading order. These estimates have low variance because they involve only lower order moments (=2), which can be obtained with better accuracy. Using the method of moments approach for direct link transmission, in [20], 4th order moments of the samples of Nakagami-*m* distribution are used for the estimation of the fading parameter *m*. As a representative illustration, we consider identical two-parameter (*α*, *β*) Weibull distribution for each hop of a multihop channel. The scale parameter *α* determines the scale (unit) of measurements in the range of distribution. A change in *α* compresses or expands the associated distribution without altering its basic form. The receiver dynamic range selection can be done based on this parameter and the CV value. The shape parameter *β* determines the basic form or shape of the distribution within the general family of distributions of interest. Let the transmitted data be *v*_1_, *v*_2_,…,*v*_*K*_ which is assumed to be deterministic with unit energy, and let the corresponding received samples at destination after *N* hop transmission be *w*_1_, *w*_2_,…,*w*_*K*_. From the noise filtered data, an unbiased estimator for the *p* ^th^ moment of the channel gain can be designed as

(38) $$\begin{array}{c} \hat{m_{p}}=\frac{1}{K}\sum_{l=1}^{K}w_{l}^{p}, \end{array}$$

where $\hat{m_{p}}$ denotes the estimate of *m*_*p*_. Using (14), the first and second order moments of *N*-cascaded Weibull distribution are obtained as

(39) $$\begin{array}{c} m_{1}=\alpha^{N}{\left[\Gamma \left(1+\frac{1}{\beta }\right)\right]}^{N}, \end{array}$$

(40) $$\begin{array}{c} m_{2}=\alpha^{2N}{\left[\Gamma \left(1+\frac{2}{\beta }\right)\right]}^{N}. \end{array}$$

These two moments can be empirically obtained as

(41) $$\begin{array}{c} \hat{m_{1}}=\frac{1}{K}\sum_{l=1}^{K}w_{l},\\ \hat{m_{2}}=\frac{1}{K}\sum_{l=1}^{K}w_{l}^{2}. \end{array}$$

Hence, the empirical coefficient of variation CV_*e*_ computed from the received data is

(42) $$\begin{array}{c} \text{C}{\text{V}}_{e}=\sqrt{\frac{\hat{m_{2}}}{{\hat{m_{1}}}^{2}}-1}. \end{array}$$

The population CV obtained earlier in (21) is

(43) $$\begin{array}{c} \text{CV}=\sqrt{\frac{\Gamma {\left(1+2/\beta \right)}^{N}}{\Gamma {\left(1+2/\beta \right)}^{2N}}-1}. \end{array}$$

Note that CV is a function of *β* only and is independent of *α*. This population CV can be tabulated for various values of *β* and the hop count *N*. In fact, Figure 2 if viewed in linear scale represents this and therefore can be used as a reference table. CV_*e*_ can be equated to CV values (in Figure 2) for a given *N*, and the corresponding $\hat{\beta }$ can be obtained. Using this estimated $\hat{\beta }$ and (39), *α* can be estimated as

(44) $$\begin{array}{c} \hat{\alpha }=\frac{{\hat{m_{1}}}^{1/N}}{\Gamma \left(1+1/\hat{\beta }\right)}. \end{array}$$

Scale parameter *α* determines the scale (unit) of measurements in the range of distribution. A change in *α* compresses or expands the associated distribution without altering its basic form. The receiver dynamic range selection can be done based on this parameter and the CV value.

### 6.2. Simulation and Numerical Results

In this section, we provide numerical and simulation results for the proposed estimators for channel parameters and hop count. The samples of Weibull fading channel coefficients for each hop were generated [21], for fixed values of *α* and *β* parameters, from which the product channel coefficients were developed according to the order of cascade. Specifically, we have generated samples with fixed parameter values of *β* = 3 and *α* = 1. The Weibull samples were verified for the mean and variance as per the standard equations. For the above parameter values, sample mean and analytical mean were obtained as 0.8935 and 0.8940, respectively. Similarly, a sample variance of 0.1050 and an analytical variance of 0.1053 were obtained. This shows the accuracy of the samples generated. 10^6^ data samples were generated for each simulation corresponding to the various hop counts.

The distribution parameters were estimated using the empirical moments and CV_*e*_ for various hop counts and the results are presented in Table 1. For example, from the generated Weibull samples for *N* = 1, the first and second order moments were computed (using (41)) as $\hat{m_{1}}=0.8935$ and $\hat{m_{2}}=0.9036$. Substituting these values in (42) gives CV_*e*_ as 0.3631. Equating this to the population CV as given in Figure 2, the corresponding $\hat{\beta }$ is obtained as 3.0001. Comparison of Table 1 and Figure 2 reveals that the empirical coefficient of variation CV_*e*_ computed from sample moments is very close to the population measures. Also, these values increase with the number of hops. Using the estimated moment $\hat{m_{1}}=0.8935$ and $\hat{\beta }=3.0001$ in the estimator given by (44) yields $\hat{\alpha }=1.0006$. Similarly, for other hop counts, product of samples is found first and the respective empirical moments, CV_*e*_ and the estimated parameters are computed and tabulated in Table 1. To evaluate the performance of the estimators, for *β* and *α*, normalised deviation (ND) is computed. For each hop count, the total deviation (TD) of the estimates is computed from the ND of each parameter as

(45) $$\begin{array}{c} \text{TD}=\left|\frac{\hat{\beta }-\beta }{\beta }\right|+\left|\frac{\hat{\alpha }-\alpha }{\alpha }\right|. \end{array}$$

The total deviation of the estimated shape and scale parameters for each order of cascade is calculated and tabulated in Table 2. It is found that the maximum TD is 7 × 10^−4^ only. This means that in the worst case the estimated parameters are within 0.07% of their actual values. It is evident that the CV value is sensitive to hop count *N*, and hence for a fixed *β* and CV value an optimum value of *N* is obtained from the CV plot.

## 7. Conclusion

Exact analyses for the characterisation of compound channels in nonregenerative cooperative relay transmissions are carried out in this work. The proposed techniques provide effective tools to assess the statistical properties of Weibull compound channel models in a convenient way. This analytical approach has been successfully pursued upon for the evaluation of a more realistic, heterogeneous compound channel model, having distinct distribution for each link, that adequately depicts the variations of the environment for a multihop wireless communication system. In situations where none of the known distributions seem to adequately fit the observed data, a better fit can be obtained by considering the proposed approach to the channel modeling. The statistical properties of the cascaded channels are investigated by computing the mean, coefficient of variation (CV), amount of fade (AF), and other higher order moments. The moments were computed using Mellin transform and it was found that the method can be conveniently applied for cases where analytical determination of the MGF is hard or even impossible. The computed measures such as CV and moments are of great importance in the design and validation of multihop wireless receivers with diversity combiner. Another unique feature of the proposed method is the computation of moments of SNR using Mellin transform techniques, which can be conveniently extended to combinations of other distributions without using the method of pdf transformation. A comparison of the channel gain and SNR variability for homogeneous and heterogeneous channel models is also carried out, which can be effectively used for assessing the distribution and selection of branches in a diversity combining receiver. The proposed estimators for distribution parameters are demonstrated to have good accuracy and can be used for the calibration of the density function from the observed samples and selection of appropriate hop count for a particular terrain.

## Figures and Tables

**Table 1:** Computation table for CV_*e*_, $\hat{\beta }$, and $\hat{\alpha }$.

*N*	$\hat{m_{1}}$	$\hat{m_{2}}$	CV_*e*_	$\hat{\beta }$	$\hat{\alpha }$
1	0.8935	0.9036	0.3631	3.0001	1.0006
2	0.7983	0.8176	0.5319	2.9998	1.0006
3	0.7103	0.7326	0.6723	2.9999	0.9995
4	0.6357	0.6623	0.7992	3.0000	0.9999
5	0.5704	0.6050	0.9269	3.0001	1.0009

**Table 2:** Deviation of the parameter estimates.

*N*	ND of $\hat{\beta }$	ND of $\hat{\alpha }$	TD
1	0.00003	0.00060	0.00063
2	0.00010	0.00060	0.00070
3	0.00003	0.00050	0.00053
4	0.00000	0.00010	0.00010
5	0.00003	0.00020	0.00023
